# The Effects of Ozone on Visual Attraction Traits of *Erodium paularense* (Geraniaceae) Flowers: Modelled Perception by Insect Pollinators

**DOI:** 10.3390/plants10122750

**Published:** 2021-12-14

**Authors:** Samuel Prieto-Benítez, Raquel Ruiz-Checa, Victoria Bermejo-Bermejo, Ignacio Gonzalez-Fernandez

**Affiliations:** Ecotoxicology of Air Pollution, Environmental Department CIEMAT, 28040 Madrid, Spain; Raquel.RuizCheca@ciemat.es (R.R.-C.); victoria.bermejo@ciemat.es (V.B.-B.); ignacio.gonzalez@ciemat.es (I.G.-F.)

**Keywords:** pollination syndrome, biodiversity, Mediterranean mountain ecosystems, petal pigments, petal area, flower spectral reflectance

## Abstract

Ozone (O_3_) effects on the visual attraction traits (color, perception and area) of petals are described for *Erodium paularense*, an endangered plant species. Plants were exposed to three O_3_ treatments: charcoal-filtered air (CFA), ambient (NFA) and ambient + 40 nL L^−1^ O_3_ (FU+) in open-top chambers. Changes in color were measured by spectral reflectance, from which the anthocyanin reflectance index (ARI) was calculated. Petal spectral reflectance was mapped onto color spaces of bees, flies and butterflies for studying color changes as perceived by different pollinator guilds. Ozone-induced increases in petal reflectance and a rise in ARI under NFA were observed. Ambient O_3_ levels also induced a partial change in the color perception of flies, with the number of petals seen as blue increasing to 53% compared to only 24% in CFA. Butterflies also showed the ability to partially perceive petal color changes, differentiating some CFA petals from NFA and FU+ petals through changes in the excitation of the UV photoreceptor. Importantly, O_3_ reduced petal area by 19.8 and 25% in NFA and FU+ relative to CFA, respectively. In sensitive species O_3_ may affect visual attraction traits important for pollination, and spectral reflectance is proposed as a novel method for studying O_3_ effects on flower color.

## 1. Introduction

Tropospheric ozone (O_3_) is one of the most important atmospheric pollutants, owing to its wide distribution and high toxicity, and it is also a short-lived greenhouse gas [1,2,3,4]. The main precursors of O_3_ are due to industrial activity and transportation. However, high O_3_ levels are found in rural, background and mountainous areas downwind of pollution sources [5,6,7,8,9].

The Mediterranean basin is considered a global biodiversity hotspot that is particularly affected by O_3_ pollution [10,11]. The characteristics of the Mediterranean climate enhance the formation and persistence of O_3_, resulting in frequent and high-concentration episodes [4,8,12,13].

One of the most adverse threats of O_3_, from a biodiversity and ecosystem functioning conservation perspective, is its effect on the reproductive ability of wild herbs [3,11,14]. O_3_ effects on flower number and biomass have been used to define critical levels for O_3_ risk assessment on semi-natural vegetation (*sensu* [15]) communities within the UNECE Convention on Long-Range Transboundary Air Pollution [16,17]. High levels of O_3_ can alter the development of flowers including timing of flowering [18,19]. Ozone can also decrease the number or biomass of flowers [20,21,22,23,24,25,26,27], although in some species it may have the opposite effect [28,29,30].

In contrast to flower number or biomass, the effects of O_3_ on other important pollination syndrome traits, such as floral traits related to the attraction of a specific group of pollinators [31,32], are less studied. Ozone effects at environmentally relevant concentrations on pollen and nectar fresh weight or composition, important nutritional sources for pollinators, have been described for some plant species [33,34,35]. Pollinators associate these nutritive rewards with a range of flower traits that can also be affected by O_3_. Ozone effects on nectar composition in *Vicia faba* changed honeybee olfactory associations, which could have an effect on floral visitation rates [35]. Floral scent blends were modified by O_3_ exposure in *Brassicaceae* species [36], while different studies show that floral volatile emissions can decompose faster in O_3_ rich atmospheres, decreasing the range of attraction for pollinators [37,38,39,40]. All these effects can result in the altered behavior of some pollinator guilds, as described for *Sinapis arvensis* plants exposed to O_3_ [28]. 

The detection of flowers also depends on the color and on the size of the colored parts [41]. Ozone has been described to decrease color in flowers of *Saintpaulia lonantha* [42], but changes in flower detectability by pollinators may vary depending on the guild of pollinators. Bees, flies and butterflies have different photoreceptors with different color sensitivities [41,43,44]. Thus, color changes and detectability by different pollinator guilds must be addressed together with studying the effects on pollination syndromes. No O_3_ effects on petal size have been reported to date.

**Figure 1:**
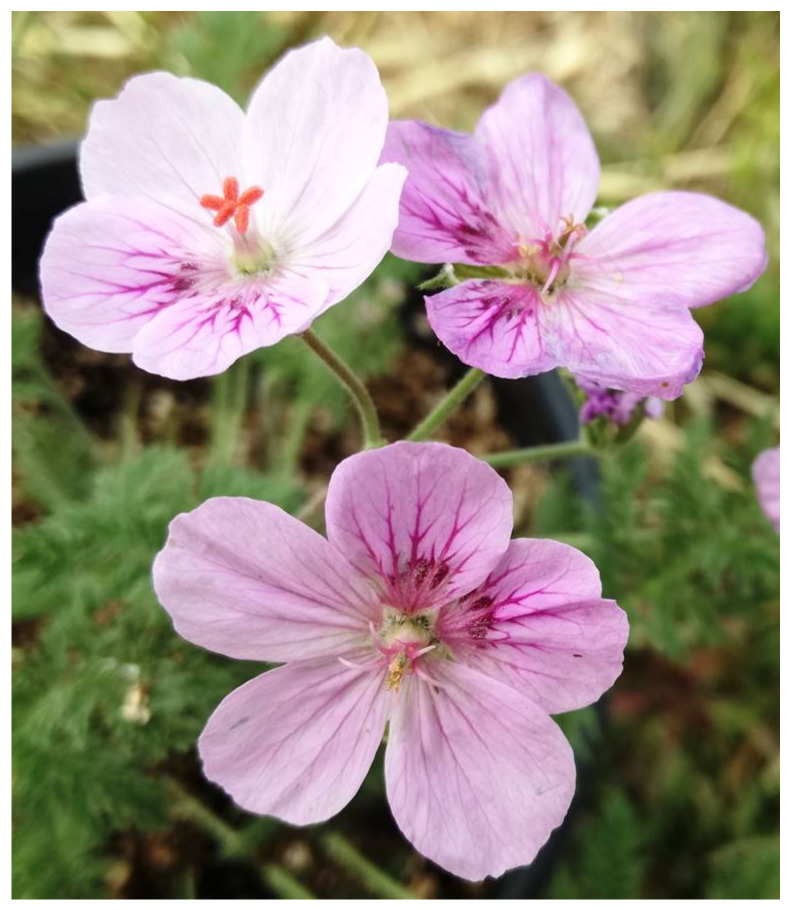
Picture of *E. paularense* in bloom.

While most of the studies about the O_3_ sensitivity of herbaceous semi-natural vegetation species have focused on widely distributed species or plant communities, relevant information for biodiversity conservation may be obtained from studies with endemic or vulnerable species. In the present experiment we studied the sensitivity of *Erodium paularense* Fzed. Gled. and Isco (*Geraniaceae*) (Figure 1), a plant endemic to central Spain that inhabits the mountain slopes of the Central System [45,46] within the Mediterranean biogeographical region. This species is categorized as endangered (EN) by the red list of Spanish vascular flora [47]. *E. paularense* is a perennial woody rosulate chamaephyte. Flowers have five purplish-pink to whitish-pink colored petals, with three to eight flowers clumped in each inflorescence, and are protandrous with visible nectar production [45,48]. *E. paularense* is a self-incompatible species; its seeds are only formed from crosspollination, and its flowers are visited by bees, butterflies and flies [48,49]. Its distinctive colored petals can be a visual signal for pollinator attraction [45]. Therefore, changes in color and petal size may affect the pollination success of this species.

The O_3_ sensitivity of *E. paularense* pollinator visual attraction traits was tested in an O_3_ fumigation experiment using open-top chambers (OTCs). Petal area and spectral reflectance were measured as treatment responses. We hypothesized that O_3_ could reduce petal development and induce color changes as a result of increases in petal anthocyanin levels, as estimated using spectral reflectance measurements, which may be related to an antioxidant response against O_3_ injury [50]. Petal reflectance was also mapped onto color spaces based on the relative excitations of the photoreceptors of bees, flies and butterflies [44] to assess if color change could be perceptible by pollinators. Specifically, we aimed to answer the following questions: (i) Is petal area affected by O_3_ exposure? (ii) Do the petal color and anthocyanin content change in response to O_3_? (iii) Does the chamber effect change the size and color of petals? (iv) Can O_3_-induced petal color changes be perceived by bees, flies and butterflies?

## 2. Results

### 2.1. Ozone Exposure and Growing Conditions

Plants growing in OTCs were subjected to a range of O_3_ exposures. Plants in charcoal-filtered air (CFA) were the least exposed to ozone, showing a filtration efficiency of 47.5% compared to the ambient O_3_ levels recorded in chamber-less plots (AMB) (Table 1A). AMB and non-filtered air (NFA) OTCs exhibited comparable O_3_ exposures, with 2.9% lower O_3_ concentration in NFA compared with AMB; non-filtered air supplemented with 40 nL L^−1^ of O_3_ (FU+) plants were the most exposed, with O_3_ concentrations 56.4% higher than those of the ambient levels and an AOT40 around 10,000 nL L^−1^ h higher than that of AMB and NFA.

During daylight hours, the OTC effect was characterized by a 3.3 °C temperature warming, 4% higher relative air humidity, a 36% increase in VPD and 17% less photosynthetic active radiation (PAR) (Table 1B). The similarity between ozone levels in AMB and NFA treatments allowed for testing the effect of the change in growing conditions on petal variables, which we termed the chamber effect. As the plants growing inside the OTCs shared the same growing conditions, the effect due to different ozone levels was tested using only OTC treatments (CFA, NFA and FU+).

### 2.2. Ozone and Chamber Effects on Petal Area, Reflectance and ARI

**Figure 2:**
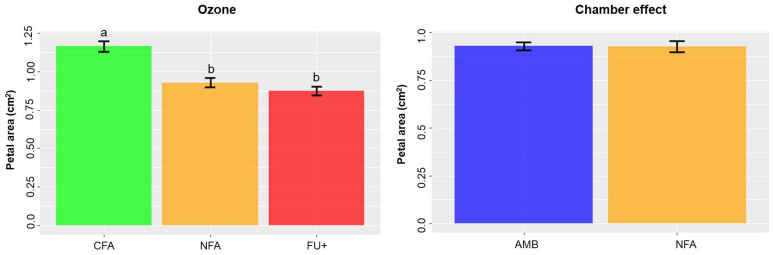
Mean and SE of petal area in charcoal-filtered air (CFA), non-filtered air (NFA), non-filtered air with 40 nL L^−1^ of O_3_ (FU+) and chamber-less (AMB) treatments. Ozone effects (CFA, NFA and FU+) and chamber effects (AMB versus NFA) are presented separately. Different letters denote differences at *p* < 0.05 among treatments.

Petal area (Figure 2) was reduced by O_3_ exposure by 19.8% and 25% in NFA and FU+ compared to CFA, respectively (χ^2^_1_ = 5.04; *p* = 0.025). The model with the plant as a random factor yielded the lowest AIC values and the priori contrast for petal area used was CFA different from NFA and FU+.

**Figure 3:**
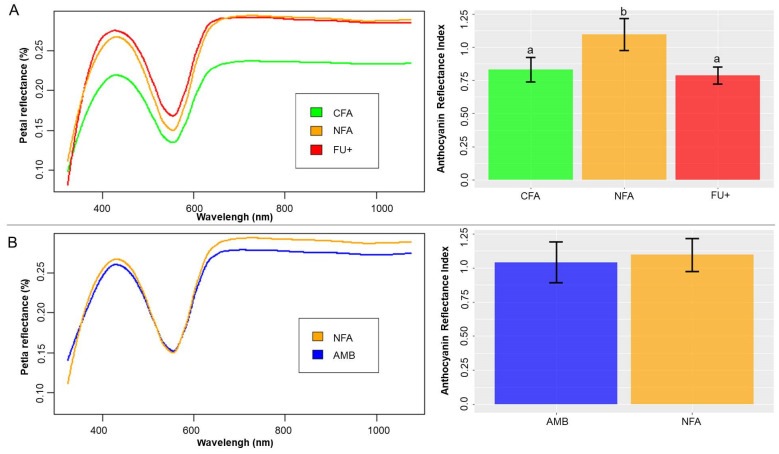
Mean petal relative reflectance spectrum per treatment (**left**) and mean anthocyanin reflectance index (ARI) of petals (**right**). (**A**) Ozone (O_3_) treatment levels are compared for charcoal-filtered air (CFA), non-filtered air (NFA) and non-filtered air with 40 nL L^−1^ of O_3_ (FU+). (**B**) Chamber effect and plants growing outside open-top chambers (AMB) are compared with NFA. Error bars denote the standard error of the mean. Different letters denote differences at *p* < 0.05 among treatments.

Ozone (NFA and FU+) also induced an increase in relative reflectance across the full spectrum (Figure 3A), and this increase was significant in the PERMANOVA analysis (F_3, 66_ =3.24; *p* = 0.02). In consequence, when the maximum height of the peak from 325 to 600 nm was analyzed, the a priori test showed a linear increase in this peak from CFA to FU+ F_2,45_ = 7.01; *p* = 0.002). However, flowers grown under CFA and FU+ shared similar ARI levels, and only NFA had high ARI values using the a priori contrasts based on treatment means (F_1,46_ = 4.2; *p* = 0.046). For ARI and the maximum height of the peak from 325 to 600 nm ozone analyses, the model with lower AIC values was the one without the plant as a random factor.

The chamber effect (AMB vs. NFA, Figure 2 and Figure 3B) did not result in changes in maximum height of peaks from 325 to 600 nm, ARI or petal size (χ^2^_1_ = 0.035; *p* = 0.85, χ^2^_1_ = 0.2; *p* = 0.88, F_1,30_ = 0.003; *p* = 0.96, respectively). For the chamber effect analyses of ARI and the maximum height of the peak from 325 to 600 nm, the model with lower AIC values was the one with the plant as a random factor, but for petal area the lowest AIC was obtained for the model without the factor plant.

### 2.3. Ozone and Chamber Effects on Pollinator Color Perception 

**Figure 4:**
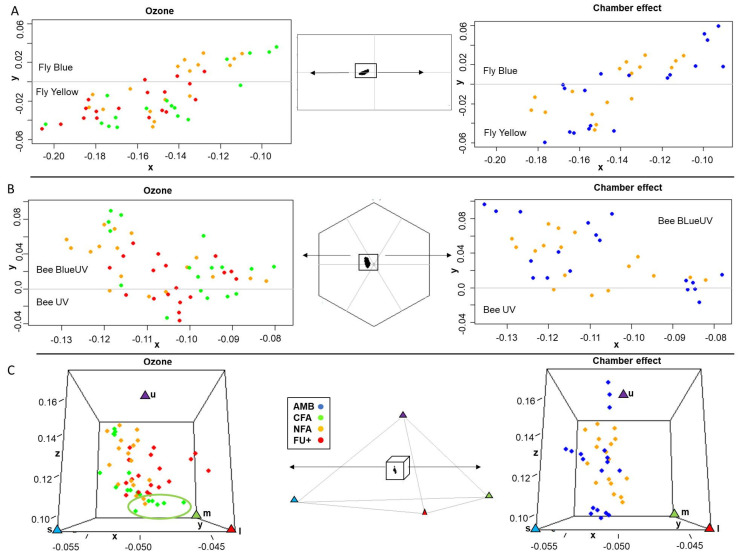
Fly (**A**), bee (**B**) and butterfly (**C**) vision of *E. paularense* petals. For each pollinator guild, petal color is represented by the color space (colors spaces in Figure 5) according to how they would be perceived. Left panels, zoom-in O_3_ treatments, charcoal-filtered air (CFA), non-filtered air (NFA) and non-filtered air + 40 nL L^−1^ of O_3_ (FU+). Right panels represent the chamber effect, and chamber-less plots (AMB) are compared with NFA. Bee UV-Blue and Bee Blue are segments of the bee hexagon. Fly Blue and Fly Yellow are fly color categories. Butterfly colors are represented by UV (u), Blue (s), Green (m) and Red (l) triangles in the x, y and z axes. For each color space, the axes values denote the excitation of each petal on each photoreceptor type scaled into the color space.

**Figure 5:**
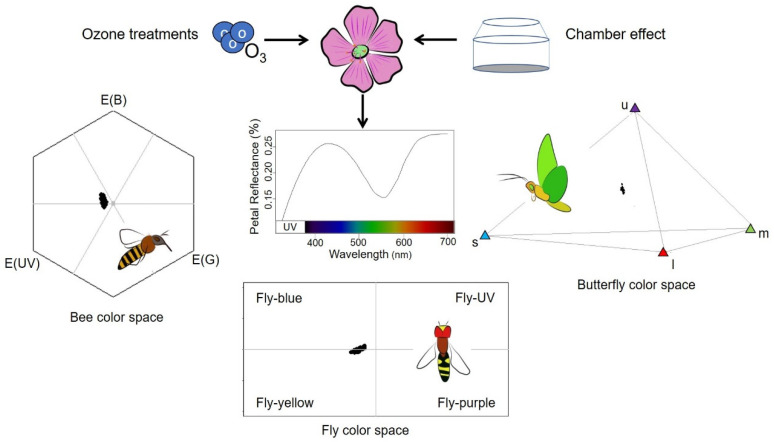
Schematic representation of the O_3_ treatment and chamber effects on *Erodium paularense* petals area and their reflectance spectrum, and how pollinators perceive petal colors. At the top, from left to right, a molecule of ozone, a flower of *E. paularense* and an OTC are presented. In the center there is the mean reflectance spectra of all the petals measured in the experiment. Around the spectra there are the color spaces for bees (hexagon), flies (rectangle, a hoverfly is represented) and butterflies (tetrahedron). The dots within the color spaces are the perceived color for each petal from the experiment. E (B), E (UV), E (G) are Blue, UV and Green bee colors. Fly Blue, Fly UV, Fly Purple and Fly Yellow are the fly color categories. Butterfly colors are UV (u), Blue (s), Green (m) and Red (l).

Figure 4 summarizes treatment effects on fly, bee and butterfly color perceptions of petals. Each dot represents the color perception of a single petal by a specific pollinator. The different axes defining the color spaces represent the relative excitation level of a particular photoreceptor. The color spaces (described in Figure 5) are divided in 4 and 6 sections for flies and bees, respectively, each of them corresponding to a perceived color. Butterfly color space is represented by a tetrahedron where each vertex corresponds to the perception of a specific color. There were some differences in the fly color space among O_3_ treatments (Figure 4A). In fly vision, most of the FU+ petals (83%) were seen as Fly Yellow, while 53% and 24% of the NFA and CFA petals, respectively, were in the Fly Blue quadrant. In bee color space, *E. paularense* petals were in the UV-Blue and Blue segments of the hexagon. Although most of the CFA petals were concentrated next to the higher values of the bee UV-Green color axis, there was no point beyond the 0.09 Euclidean distance threshold that would indicate a perceptible change in color for bees (Figure 4B). In butterfly vision, the UV photoreceptor (u) showed the greatest variability among O_3_ treatments, with most CFA petals situated in the lower values of the UV photoreceptor. Although considerable overlapping was found between treatment levels, six CFA petals exceeded the 0.03 threshold established for the color discrimination of butterflies (ellipse in Figure 4C) in the UV axis (photoreceptor u). A total of 47 and 33% of NFA and FU+ petals, respectively, showed a perceptible color change for butterflies compared with CFA petals highlighted by the ellipse. There was no chamber effect in the vision of the *E. paularense* petals, and no clear differentiation between AMB and NFA treatments were detected in any of the three color spaces (Figure 4).

## 3. Discussion

The main objective of this study, testing the use of petal area and spectral reflectance for the description of O_3_ effects on flower visual attractiveness, shows that both variables may be affected by O_3_ exposure. These two parameters related with pollinator visual attraction can be used in combination with other pollinator attraction variables such as olfactory attraction and nutritional rewards in order to describe the potential mechanisms of the O_3_ effect on pollination. Since this study is a first approach to studying O_3_ effects on visual pollinator attraction, the potential importance of visual signaling on pollinator attraction and ways for future research and limitations of the present study are further discussed.

Importantly, from the pollinator attraction perspective, petal size was affected by O_3_ exposure, with flowers developing under higher O_3_ exposures having smaller petals than those developing under CFA. Other studies have shown the negative effects of O_3_ on individual flower weight [24], number [3,16,25] and corolla length [51], but to the best of our knowledge this is the first study reporting the negative effects of O_3_ on petal size. In species where flowers are tightly grouped in inflorescences, the effect on inflorescence size may be important, but in *E. paularense* the flowers within the inflorescence are clearly separated from each other and do not open synchronously. Thus, it was considered that individual flower size would be a more relevant visual attraction trait than inflorescence size. Smaller petals are less visible, making the pollinators’ search for the *E. paularense* flower more difficult. In the presence of other generalist plant species that are less sensitive to O_3_, pollinators may be less attracted by *E. paularense* flowers, which may result in lower flower visitation frequency and lower reproductive success. In this way, it has been observed that changes in the color and size of petals in *Moricandia arvensis* can result in shifts of pollinator guilds [52]. These types of effects can be particularly important for the reproductive success of *E. paularense*. Low viable seed production rates have been reported for this species, which are not limited by pollen availability [48]. Low viable seed production rates may be related with the provenance of the pollen, with low-quality pollinators visiting more flowers within than among plants and producing self-incompatibilities [53]. Thus, O_3_ effects on pollinator attraction traits might be of particular importance for this species.

Ozone-induced changes in petal reflectance, the maximum height of reflectance peaks and ARI values could be associated with changes in petal anthocyanin levels. Anthocyanins are the main pigments present in blue and purple flowers [54], which are produced by petal epidermis cells during flower development [55]. High anthocyanin concentration found in the petals of the related species *Erodium cicutarium* [56] have been associated with minimum and maximum reflectance peak values around 550 and 650 nm [57,58,59,60], which are also present in *E. paularense*. The similarity of the reflectance spectra of both species suggest that anthocyanin could also be the main pigment of *E. paularense* petals and the reported changes in petal reflectance induced by O_3_ may be related to changes in anthocyanin content. 

The synthesis of anthocyanin in leaves has been described as an antioxidant response against abiotic stresses produced by low temperature, UV, drought, heavy metals or wounding [59,61]. Regarding O_3_ stress, most of the existing studies show that this pollutant can also induce an increase of foliar anthocyanin levels as an early antioxidant response [50,62,63,64,65]. We hypothesized that O_3_ may be directly absorbed by the stomatal pores present in petals of most plant species [66], triggering an antioxidant response that results in increased anthocyanin production. However, the response to O_3_ exposure in this experiment was not linear, as has been reported for other species and response parameters [10]. Further experiments would be necessary to identify the possible mechanisms explaining O_3_ effects on petal pigment levels. 

The changes found in the reflectance spectra did not result in a strong color perception change, an important pollinator attraction trait, for the three pollinator guilds evaluated, but some subtle effects were observed. Petals were within the UV-Blue or Blue segment of the bee vision hexagon, and none were further than the minimum Euclidean distance required to be perceptible as a color change for bees. On the contrary, in flies, most of the FU+ petals were perceived as yellow, while some petal under less ozone-dense levels (NFA and CFA) were perceived as blue. Additionally, butterflies seem to be able to distinguish some O_3_-induced changes in petal color. Most of the petals under CFA had lower values for the UV photoreceptor than ambient (NFA) or elevated (FU+) O_3_ treatments. The visual perception of petals by pollinators does not only depend on pigment concentration but also on other traits such as petal epidermis micromorphology [67]. Future studies could also address these effects since O_3_ has been described as capable of affecting leaf epidermis micromorphology (e.g., [68]). Further research will be needed to study if ozone effects on visual attraction traits are accompanied by changes in pollinator rewarding, which may result in pollinator behavioral changes. Beyond the study of the mechanisms by which ozone may affect pollinator attraction plant traits, other experiments such as bioassays or pollinator visitation rate monitoring would improve the understanding of the potential consequences of all of these changes on the actual behavior of pollinators. 

The use of OTCs to study O_3_ effects on plants has the advantage of controlled O_3_ exposures under close to field growing conditions and allowing exposure to below ambient O_3_ levels in the CFA treatment. Environmentally relevant ozone exposures have been applied in this experiment. Plants under high-O_3_-level treatment (FU+) were exposed to levels comparable with the highest concentrations recorded in the natural distribution area of *E. paularense* at *Sierra de Guadarrama* in central Spain during dry and warm years [5]. The maximum monthly average O_3_ concentrations recorded at the mid altitudes of the Sierra de Guadarrama reached up to 55 nL L^−1^, which are comparable to the average value at the elevated O_3_ treatment used in this study (Table 1). However, OTCs change, by its design, plant growing conditions (Table 1), which could have modified the pollinator syndrome traits of *E. paularense*. In this experiment, plants growing in OTCs received less light than plants growing outside OTCs, although OTCs plants also received direct sunlight through the open top of the chamber for several hours a day. OTCs also changed the VPD conditions, but VPD levels remained in non-limiting levels of gas exchange for a range of species [69], both inside and outside OTCs, and it was not expected to have a limiting effect on plant growth and flower development. The main chamber effect recorded was a mean temperature increase of 3 °C, and an increase of 5 °C in absolute maximum temperature. It has been proven that different traits of pollination syndrome are affected by temperature increases. Higher temperatures may produce changes in species phenology, advancing the flowering time and producing temporal mismatches between the pollinators and flower presence, lowering the reproductive plant fitness [70,71]. Temperature increases can also affect the plant–pollinator interaction by changing the scent production and nectar composition, decreasing the pollen amount and reducing the flower size [71]. Some studies also indicated that high temperatures decreased the amounts of anthocyanins in flower petals of other species [72,73,74]. However, in the present study, temperature increases in OTCs had no effect on the petal area of *E. paularense* nor the reflectance spectra or the amounts of anthocyanins in petals as evaluated with ARI compared with AMB plants.

## 4. Materials and Methods

### 4.1. OTC Experiment

The experiment was performed in an NCLAN-type OTC (adapted from the original design by [75]) facility located in central Spain, 40°3′ N, 4°26′ W, 450 m.a.s.l. The experiment was conducted in 4 plots with three O_3_ treatments and one ambient plot (AMB) without chamber. Three O_3_ treatments were applied: charcoal-filtered air (CFA), non-filtered air (NFA) reproducing ambient levels and non-filtered air supplemented with 40 nL L^−1^ of O_3_ (FU+). Ozone was produced from pure O_2_ by means of an O_3_ generator (A2Z Ozone, Inc., Louisville, KY, USA) and supplied to the FU+ plot 8 h day^−1^ (7:00 to 15:00 GTM) over 37 days. Ozone concentrations inside each chamber and AMB plots were monitored continuously using an UV absorbance monitor (ML^®^ 9810B, Teledyne, Thousand Oaks, CA, USA) with an automated time-sharing system. Ozone exposure was described by the mean (7–15 h) concentration and the AOT40 index, calculated as the accumulated hourly concentration over a threshold of 40 nL L^−1^ during daylight hours over the fumigation period. Meteorological conditions (air temperature—T (°C), air relative humidity—RH (%) and photosynthetic active radiation—PAR (µmol m^−2^ s^−1^)) inside and outside the OTCs were continuously measured using AM2315 (T and RH; Adafruit Industries LLC, New York, NY, USA) and Apogee SQ 110 (PAR; Apogee Instruments, Inc., Logan, UT, USA) sensors. The plots were not in the shadow of any vegetation or building. The OTCs are constructed of the same material and have the same shape. Therefore, apart from the different ozone concentration, the conditions were the same inside the chambers. Differences in meteorological conditions between ambient and OTC plots were used to describe the chamber effect on measured variables. The AMB treatment was considered as control when assessing the chamber effect within the OTCs.

### 4.2. Plant Material

*Erodium paularense* plants were raised from seeds collected from natural populations at the *Sierra de Guadarrama* National Park and transplanted to 21 pots containing peat, vermiculite and perlite (60:20:20) in the spring of 2017. Plants grew outdoors, except during O_3_ fumigation experiments, and were kept well irrigated and fertilized. In the second spring growing season, plants were exposed to O_3_ in OTC for 70 days. AOT40 values for each of the treatments were 5920, 12, 4324, 16761 nL L^−1^.h for AMB, CFA, NFA and FU+, respectively.

The present study was conducted in the third spring growing season on the same plant material. The two-year-old plants were transferred to the OTCs on 3 April and were exposed to the same O_3_ fumigation treatments as the previous year for 37 days. Prior to the start of the O_3_ fumigation treatments all the inflorescences were removed, so that the measurements were conducted on flowers developed under the treatment conditions described in Table 1.

### 4.3. Spectral Reflectance Measurements, Anthocyanin Index and Petal Area

On 10 May 2019, for each of two plants per OTC in the same phenological stage, three petals from three fully developed flowers growing in different inflorescences were excised for measurements. Right after the excision, alternating between the different O_3_ treatments, petal spectral reflectance measurements were collected. Relative reflectance spectra of each petal were measured with a Hand-Held FieldSpec spectroradiometer (Analytical Spectral Devices, Boulder, CO, USA) on the 325 nm to 1075 nm wavelength range with a spectral resolution of 3 nm. Petals were inserted in a leaf clip illuminated with a halogen bulb (2900 K color temperature) and measured against a black background (black painted vinyl). A white reference (Gore-Tex white PTFE reflector material) was taken before each measurement. An average of 25 spectra per petal were considered for analysis. 

Ozone and chamber effects on petal spectral reflectance were evaluated for the full range of spectral reflectance wavelengths (325–1075 nm), the maximum height of spectral peaks and the anthocyanin reflectance index (ARI). Maximum height of peaks from 325 to 600 nm was extracted from each reflectance spectra in R software version 4.0.2 [76] using the function *peakshape* (*Pavo* package) [77]. The content of anthocyanins in each petal was estimated with the anthocyanin reflectance index, ARI = (R800/R550) − (R800/R700) [78], where R is the relative reflectance value at the specified wavelength. 

After reflectance measurements, metric-scaled photos were taken on all intact petals, and the area was measured using ImageJ software [79]. A total of 70 and 66 petal measurements were collected for spectral reflectance and petal area, respectively (Table 1A).

### 4.4. Pollinator Color Perception

*Pavo* package [77] in R software was used to analyze the reflectance spectra and to plot the color space of bees, flies and butterflies using available models in the wavelength range of 300–700 nm. To generate the visual model, both the petal spectral and sensitivity data were trimmed to the 325–700 nm wavelength range. Visual models for each of bees and flies were generated based on the spectral sensitivity data of each photoreceptor class, 3 and 4 classes, respectively, already implemented in *Pavo* package. Butterfly sensitivity data of 4 photoreceptor classes were extracted from [44] (see also [80]). Similar to [44], the following setups for bee, fly and butterfly visuals models were chosen: green foliage as background spectra and D65 (standard day light) as illuminant. The longest-wavelength photoreceptor of each pollinator was used to calculate luminance (achromatic) receptor stimulation [77]. 

Hexagon, rectangle and tetrahedron plots were performed to represent the color space of bee, fly and butterflies, respectively [43,44,81]. Figure 5 summarizes how all petal spectra were represented on each color space. To assess the perceptible color change, we followed the criteria established by [44] to ensure at least 60% of correct color discrimination. Flies see different colors when stimuli are in different color space quadrants. For bees and butterflies a minimum distance in the color space axis values of 0.09 and 0.03 was required, respectively [44].

### 4.5. Statistics

Two sets of analyses were performed, one to study the influence of O_3_ treatments (excluding AMB plot) and a second one to assess the chamber effect (NFA vs. AMB plots) on ARI, maximum height of peaks and petal area. Petal area was used as covariable for ARI and reflectance analyses as petals did not completely cover the background surface of the spectroradiometer, and this may affect the comparison of relative reflectance values among treatments. Then, to control for the plant-to-plant variability in petals traits, the identity of each plant was considered as a random factor. For each analysis, the AIC (Akaike information criterion) value was used to choose the most parsimonious model between lineal (without random factor) or mixed lineal model (with random factor). Ozone effects were evaluated using increasing linear a-priori contrasts as expected. However, contrasts comparing O_3_ treatment means were also tested. For petal area, a reduction in size was expected, thus linear decreasing and difference from the control treatment a-priori contrasts (CFA versus NFA or FU+) were used for this variable. To fulfill the assumption of normality of the residuals for ANOVA, a logarithmic transformation of ARI was performed. A-priori contrasts in linear models were based on [82]. Mixed linear models were performed using *lmer* function (lme4 package; [83]). *Anova* function was used for the mixed models (car package; [84]). To test the hypothesis of a “linear increase” the *contr.poly* function [76] was used. For ARI, the contrast coding based on the treatment means was CFA = −1, NFA = 2 and FU+ = −1. The contrast coding for the “difference from control treatment” hypothesis was CFA = 2, NFA= −1, FU+ = −1 for petal area. One-way ANOVA tests were performed to evaluate the chamber effect (NFA vs. AMB) on ARI, maximum height of peaks and petal area.

A PERMANOVA analysis was performed to compare the reflectance spectra values (from 325 to 1075 nm) as a multivariate trait among treatments. To prevent PERMANOVA from being largely influenced by high reflectance values at some wavelengths, data were squared then root transformed, and a similarity matrix was made by Bray–Curtis approximation [85]. A total of 9999 permutations were chosen, and PERMANOVA was performed using *vegan* package [86]. All the statistical analyses described were carried out in R software [76].

## 5. Conclusions

This study highlights that visual attraction parameters such as petal area and color may be sensitive to O_3_ exposure. These features are proposed as novel response variables for studying O_3_ effects on pollination syndrome traits associated with flower visual attraction, although the results presented warrant further research. 

This O_3_ fumigation experiment shows how petal area was reduced by O_3_ exposure in *Erodium paularense*, an endangered and endemic herbaceous species of the The Central System in Spain. Petal color, as perceived by different pollinator guilds, was partially modified for butterflies and flies, and indications of a non-linear O_3_ effect on petal anthocyanin content were suggested by experimental results. Current high O_3_ levels found in the distribution area of this species are high enough to potentially affect pollination syndrome traits as described in this study, imposing an additional burden to climatic warming. Other pollinator syndrome traits may also be affected in this species. Further studies would be needed in order to clarify the overall effect of O_3_ on the pollination success and survival of this species before this threat can be addressed in the conservation management policies of *E. paularense*. 

## Figures and Tables

**Table 1:** (**A**) Ozone exposure conditions during the experiment. N LM or LMM and N PERM are the number of petals used in lineal models or lineal mixed models and PERMANOVA, respectively. AOT40 is the accumulated (37 days) O_3_ concentration over a threshold of 40 nL L^−1^ during daylight hours, 7–15 h. O_3_ nL L^−1^ is the mean (7–15 h) O_3_ concentration. Daily O_3_ mean nL L^−1^ is the mean (0–24 h) O_3_ concentration. (**B**) Chamber effect on growing conditions. Mean and max temperature (Tª), relative humidity (RH), vapor pressure deficit (VDP) and photosynthetic active radiation (PAR) are the mean values during daylight hours outside (ambient) and inside the OTCs (OTC).

**(A) Ozone Exposure and N**			**AOT40**	**7–15 h. O_3_ Mean **	**Daily O_3_ Mean**
**Treatment**	**N LM or LMM **	**N PERM.**	**(nL L^−1^ h)**	**(nL L^−1^)**	**(nL L^−1^)**
**AMB**	17	18	2922.7	41.3	36
**CFA**	17	17	23.7	21.7	17.3
**NFA**	15	17	2333.2	40.1	35.2
**FU+**	17	18	12121.5	64.6	48.8
**(B) Growth Conditions**					
	**Mean Tª (°C)**	**Max Tª (°C)**	**RH (%)**	**VPD (kPa)**	**PAR µmol (m^−2^ s^−1^)**
**Ambient**	15.8	28.6	52.5	0.78	841
**OTC**	19.1	33.6	56.5	1.06	698.2

## Data Availability

Publicly available datasets were analyzed in this study. This data can be found here: http://rdgroups.ciemat.es/web/geca-ciemat/. Averaged flower spectral data available at FReD database (http://www.reflectance.co.uk/).
